# Placental Protein 13 (Galectin-13) Polarizes Neutrophils Toward an Immune Regulatory Phenotype

**DOI:** 10.3389/fimmu.2020.00145

**Published:** 2020-02-12

**Authors:** Lenka Vokalova, Andrea Balogh, Eszter Toth, Shane V. Van Breda, Günther Schäfer, Irene Hoesli, Olav Lapaire, Sinuhe Hahn, Nandor Gabor Than, Simona W. Rossi

**Affiliations:** ^1^Prenatal Medicine, Department of Biomedicine, University and University Hospital Basel, Basel, Switzerland; ^2^Systems Biology of Reproduction Research Group, Institute of Enzymology, Research Centre for Natural Sciences, Budapest, Hungary; ^3^Department of Antenatal Care, University Women's Hospital Basel, Basel, Switzerland; ^4^First Department of Pathology and Experimental Cancer Research, Semmelweis University, Budapest, Hungary; ^5^Maternity Private Clinic of Obstetrics and Gynecology, Budapest, Hungary

**Keywords:** placental protein 13, neutrophils, preeclampsia, pregnancy, tolerance, immunoregulation, galectin, tumor permissive phenotype

## Abstract

Termed as galectin-13, placental protein 13 (PP13) is exclusively expressed in the placenta of anthropoid primates. Research on PP13 in normal and pathologic pregnancies show alteration of PP13 concentrations in pregnancy affected by preeclampsia or gestational diabetes. Galectins are also described as potent immunomodulators, and PP13 regulates T cell function in the placenta. Therefore, this study aims to investigate the effects of PP13 on neutrophils; a cell type often ignored in pregnancy, but present in the uterus and placenta from the early stages of pregnancy. Since neutrophil function is dysregulated during pathologic pregnancies, a link between PP13 and neutrophil activity is possible. We determined that PP13 reduces the apoptosis rate in neutrophils. Also, PP13 increases the expression of PD-L1 and production of HGF, TNF-α, reactive oxygen species (ROS), and MMP-9 in these cells. This phenotype resembles one observed in permissive tumor neutrophils; able to sustain tissue and vessel growth, and inhibit T cell activation. At the same time, PP13 does not alter all neutrophil functions, i.e., extrusion of neutrophil extracellular traps, degranulation, phagocytosis, and ROS production following bacterial exposure. PP13 seems to play an essential role in regulating the activity of neutrophils in the placenta by polarizing them toward a placental-growth-permissive phenotype.

## Introduction

During pregnancy, the immune system is challenged by the presence of a semi-allogeneic fetus and needs to maintain its protecting role for the mother. One key event in the formation of the placenta that keeps the fetus physically separated from the mother is the invasion of the extravillous cytotrophoblasts into maternal spiral arteries to remodel the wall of these vessels, enabling increased and continuous blood flow from mother's vascular system to the placenta to sustain fetal growth [rev. in (1)]. This process is tightly regulated, and not all of the essential players are known yet. During the last 10 years, genetic analysis revealed the presence of regulatory molecules exclusively expressed in the placenta of human primates. The quest for novel biomarkers for early identification of abnormal pregnancies revealed a novel potential candidate, placental protein 13 (PP13). PP13 is a member of the galectin family, a protein dimer produced by the trophoblast and is thought to be involved in normal placentation (2–4). The gene encoding PP13 (*LGALS13*) is localized on chromosome 19 in a gene cluster, neighbored by other galectin genes also expressed solely by the placenta. PP13 protein is secreted from a very early stage of pregnancy and can already be detected in the bloodstream of pregnant women from the 5th week of gestation (5). Immunohistochemistry and RNA hybridization studies have pointed to its predominant localization in the placental syncytiotrophoblast layer and blood vessels (3, 5). Supporting the importance of PP13 immune functions in the placenta, a growing number of studies have shown that the down-regulation of PP13 in the placenta and maternal blood is associated with the development of severe pregnancy complications with a robust immune component, such as preeclampsia (6–8) and miscarriages (9). Recently, Balogh et al. reported that PP13 increases the apoptosis of T cells and induces the production of interleukin (IL)-8 (CXCL8) in these cells (9). It is vital since IL-8 is involved in angiogenesis and is also a potent chemoattractant for neutrophils (9).

The role of neutrophils in the placenta is still a matter of debate since their presence was mostly associated with adverse pregnancy outcomes, i.e., preeclampsia (10), gestational diabetes (11), and infections [i.e., intra-amniotic infection (12, 13) and other infections rev. in (14)]. Neutrophils were shown to be involved in the exacerbation of the symptoms while releasing extracellular traps (NET), impairing the blood flow to the fetus, and increasing the level of inflammation by releasing several proteases (10, 11). Since neutrophils are present in the decidua during the first trimester of pregnancy (15), while decidual NK cells promote neutrophil migration, survival, and activation (16) [and rev. in (17)]. In addition, T cells in the presence of PP13 start producing chemoattractants to promote neutrophil extravasation to the decidua (9). PP13 also creates zones of necrosis to trap immune cells and allow trophoblast invasion and vessel remodeling (18). Thus, we decided to investigate the effect of PP13 on neutrophil biology. Indeed, neutrophils were already described in the cancer setting to sustain cancer growth, supporting angiogenesis, and inhibiting T cell activity [among others recently rev. in (19, 20)]. The immunology of the placenta was recently proposed by Mor et al. (14) as being similar to the immunology of cancer, where a tumor-growth-permissive environment is required. Some of the molecules described in cancer or sepsis that characterize the polarization of neutrophils are programmed death-ligand 1 (PD-L1) positive on their surface (21), the secretion of regulatory cytokines, i.e., IL-10 (22), polarizing cytokines, i.e., IL-4 (23) or tumor necrosis factor alpha (TNF-α) (24), enzymes able to untie junctions, i.e., matrix metalloproteinase 9 (MMP-9) (25), and growth factors like vascular endothelial growth factor (VEGF) (26) and hepatocyte growth factor (HGF) (27, 28) that directly stimulate angiogenesis. Not to be forgotten is the role of reactive oxygen species (ROS) produced by neutrophils upon activation, also described to induce angiogenesis (29) and control T cell proliferation (30). We, therefore, planned to study the potential of PP13 to polarize neutrophils toward a “growth-permissive” phenotype able to sustain trophoblast growth and invasion. We found that PP13 sustains the survival of neutrophils, induces them to produce ROS, HGF, and MMP-9, upregulates the expression of PD-L1 while maintaining their functionality, like NET formation, degranulation, phagocytosis, and bacterial killing.

## Materials and Methods

### Human Neutrophil Isolation From Peripheral Blood

EDTA anticoagulated blood was obtained from male and non-pregnant female healthy human donors at the Blood Bank of the Swiss Red Cross, Basel. Neutrophils were isolated by PolymorphPrep™ (Axis-Shield). Erythrocytes were lysed by red blood cell lysis buffer (Roche). Isolated neutrophils were resuspended in RPMI 1640 supplemented with 10% fetal bovine serum (FBS) and 1% penicillin/streptomycin (P/S) and cultured for 6–72 h depending on the experiment. All assays were repeated with multiple donors to obtain experimental replicates.

### Expression and Purification of Recombinant Galectin-13 and Control GAL

Recombinant PP13 and control galectin (Gal) that was misfolded (personal communication with NG Than) were expressed as previously described (7) with modifications. Expression plasmids containing full-length PP13 or control Gal, N-terminal maltose-binding protein (MBP)- and C-terminal His_6_-tags, were transformed into ClearColi BL21 (DE3) (Lucigen). For protein expression cells were grown in LB-Miller broth to OD_600_ = 0.6 at 37°C, induced with 0.4 mM IPTG and grown for a further 4 h at 30°C. The following purification steps were applied: affinity purification on MBPTrap HP column (GE Healthcare Life Sciences), size exclusion chromatography (Superdex 200 Increase SEC column, GE Healthcare Life Sciences) for elimination of aggregates (only for control Gal), MBP cleavage by Tobacco Etch virus (TEV) protease [expressed and purified according to Kapust and Waugh (31)], affinity chromatography on HisTrap HP columns (GE Healthcare Life Sciences), desalting and buffer exchange on Bio-Gel P-6 Desalting Cartridge (Bio-Scale Mini, Bio-Rad). All steps were carried out in the presence of 1 mM dithiothreitol (DTT). Finally, PP13 and control Gal in PBS supplemented with 1 mM DTT were aliquoted and stored at −80°C.

### Apoptosis Assay

Neutrophils (1 × 10^6^) were incubated for 24 h in tissue culture plates with 3 μg/ml recombinant PP13 or control Gal in RPMI 1640 medium supplemented with 10% FBS and 1% P/S. Third trimester hormonal environment was simulated by supplementation of the medium with pregnancy hormones (32): progesterone (P4, 100 ng/ml), estradiol (E2, 10 ng/ml), and estriol (E3, 20 ng/ml). Afterwards, cells were incubated in 100 μl annexin binding buffer containing phycoerythrin-conjugated Annexin V (Annexin V-PE) and 7-amino-actinomycin D (7-AAD) (Annexin-V Apoptosis Detection Kit, ThermoFisher Scientific) for 15 min at room temperature (RT) in the dark. After incubation, 400 μl annexin binding buffer was added and samples were measured immediately on a BD Accuri™ C6 FACS (BD Biosciences). The Annexin V-PE^+^/7-AAD^−^ and Annexin V-PE^+^/7-AAD^+^ populations were taken as measurements of early and late apoptotic cells, respectively. Data were analyzed using FlowJo v10 software (FlowJo, LLC).

### BeWo Cells and Co-culture Conditions

BeWo cells (ATCC CCL-98) were grown at 37°C under a humidified 5% CO_2_/95% in F-12K medium containing 10% FBS and 1% P/S. Experiments were performed when cells reached 80% confluency between passages 5 and 10 in 24 well plates.

For co-culture experiments BeWo cells were seeded into 24-well plates in growing medium. When cells were attached to the plate and reached 80–90% of confluency (24 h) 1 × 10^5^ of freshly isolated neutrophils were added. The co-cultures were treated with PP13 (3 μg/ml). After 24 h incubation neutrophils were collected, washed with 1 ml PBS (pH 7.4), resuspended in 50 μl staining buffer and proceeded with flow cytometry staining.

### Flow Cytometry Surface Staining

Fc receptor blocking on neutrophils was performed for 10 min (FcR binding inhibitor antibody, Invitrogen Life Technologies) in staining buffer (PBS with 5% FBS and 0.1% sodium azide) on ice. Cells were washed once and then stained with CD66b-FITC (BioLegend, clone: G10F5), CD11b-PE (BioLegend, clone: ICRF44), and PD-L1-APC (eBioscience, clone: MIH1) for 20 min on ice in dark. To measure the binding of recombinant PP13 to the surface of neutrophils, 2 × 10^5^ cells were initially washed in PBS containing 1% BSA. Recombinant PP13, which we conjugated with CF488 fluorophore using the Mix-n-Stain CF488 kit (Sigma-Aldrich) according to the manufacturers protocol, was incubated for 60 min on ice or at 37°C. After washing, Fc receptors were blocked with human FcR blocking reagent (Miltenyi Biotec) for 5 min on ice. Anti-CD66b-APC antibody (Biolegend) was used to stain neutrophils. In both cases, stained neutrophils were washed twice and then acquired on a CytoFLEX device (Beckman Coulter) by collecting data from 50,000 cells. Data was analyzed using FlowJo v10 software.

### ROS Production Assay

ROS production was performed as previously described (33). Briefly, PMNs (1 × 10^6^ cells/ml) treated or not with PP13 or control Gal (3 μg/ml) for 1 h were incubated with dihydrorhodamine 123 (DHR123). Oxidation of DHR123 to rhodamine 123 (R123) was measured by Biotek Synergy H1 Hybrid Reader (Biotek) plate reader (excitation 485 nm, emission 570 nm).

### ELISA Assays

Commercially available Human ELISA Kits for HGF (ab100534), IL-4 (ab215089), IL-10 (ab100549), MMP-9 (ab246439), and VEGF-α (ab222510) (Abcam) were used to estimate concentration of cytokines in culture medium supernatants of neutrophils treated with PP13 (3 μg/ml) for 1 h using the manufacturer's instructions.

### RNA Isolation and Quantitative Real-Time PCR

Initially, isolated neutrophils were treated with PP13 (3 μg/ml) or left untreated for 1 h. Total RNA was isolated from 3 × 10^6^ neutrophils using the RNeasy Mini Kit (Qiagen). TaqMan RT-PCR was performed utilizing the Applied Biosystems StepOne Plus cycler (Applied Biosystems) and TaqMan Gene Expression Assay primer and probe sets (Applied Biosystems) for *TNF* (HS01113624_g1), *SERPINB1* (HS00961948_m1), and *GAPDH* (HS99999905_m1).

### Chemotaxis Assay

Chemotaxis assays were performed using a 24-well transwell plate (34, 35). Briefly, PP13 and Control Gal (3 μg/ml) were diluted in RPMI 1640 containing 1% BSA, 10 mM HEPES, and were placed in the bottom wells of the chamber. Neutrophils (1 × 10^5^/well) in 150 μl medium were added to the upper wells separated by a 3 μm pore size uncoated polycarbonate membrane (Corning) from the lower wells. N-formyl-methionyl-leucyl-phenylalanine (fMLP, 100 nM) was used as a positive control and medium alone as a negative control. After incubating at 37°C for 45 min, transwell membranes and all liquid in bottom well were removed and the content of the well-stained for flow cytometry and identification of neutrophils.

### Neutrophil Elastase Activity Measurement

Neutrophil elastase (NE) activity was measured as described in (36). Briefly, 50 μL of medium supernatant was collected after 24 h culturing of neutrophils in the presence or absence of PP13 (3 μg/ml), then incubated with the elastase substrate N-methoxysuccinyl-Ala-Ala-Pro-Val-7-amido-4-methylcoumarin (0.25 mM, Sigma) in PBS for 30 min at 37°C, 5% CO_2_ in the dark. The reaction product was analyzed at 360/455 nm.

### Immunocytochemistry Analysis of NETs

NETs were quantified by immunofluorescence staining of 2.5 × 10^4^ neutrophils/well in a 96-well plate in RPMI 1640 medium. Neutrophils were seeded into plate and pre-treated with PP13 for 2 h at 37°C. Afterwards, neutrophils were stimulated by phorbol 12-myristate 13-acetate (PMA, 20 nM) and Ca^2+^-ionophore A23187 (2.5 μM) for 1 h and fixed in 4% paraformaldehyde. NETs were stained with mouse anti-human MPO antibody (1:500, ab25989, Abcam) and goat anti-mouse IgG AF555 (1:500, A21424, Invitrogen Life Technologies) (37, 38). DNA was counterstained with 4′,6-diamidino-2-phenylindole (DAPI, D9542, Sigma-Aldrich). NETs were visualized using a Nikon Eclipse TI microscope and analyzed with the NETQUANT (39).

### Neutrophil Co-culture With Heat Killed *E. coli* and *S. aureus*

Heat killed *E. coli* (ATCC 25922) or methicillin-susceptible *S. Aureus* (ATCC 29213) bacteria were incubated with freshly isolated neutrophils (10^5^ cells/well) in the presence of PP13 (3 μg/ml) at 37°C for 30 min. The neutrophils to bacterium ratio was 1:100. ROS was measured immediately after adding bacteria and after 30 min of incubation. Zero time point was used as baseline.

### Phagocytosis Assay

Neutrophils were cultured with or without PP13 for 72 h or 30 min before exposure to 40 kDa Fluorescein isothiocyanate (FITC)-dextran (1 mg/ml, Sigma). Cells were allowed to phagocyte for 60 min at 37°C. Afterwards, samples were washed with PBS and stained with the LIVE/DEAD™ Fixable Red Dead Cell Stain Kit (Invitrogen Life Technologies) for 30 min at RT. Samples were analyzed by a BD CytoFLEX instrument and data were analyzed using FlowJo v10 software. Dead cells were excluded from the analysis.

### Statistics

Data were analyzed by one- or two- way ANOVA, or Student's *t*-test using GraphPad Prism 8. Quantitative real-time PCR data (Ct-value) were normalized to GAPDH (ΔCt value) and analyzed by Student's *t*-test. Data are presented as mean with standard deviation. Values of *P* < 0.05 were considered significant (^*^P < 0.05, ^**^P < 0.01, ^***^P < 0.001).

## Results

### PP13 Increases the Survival of Neutrophils in Culture

**Figure 1:**
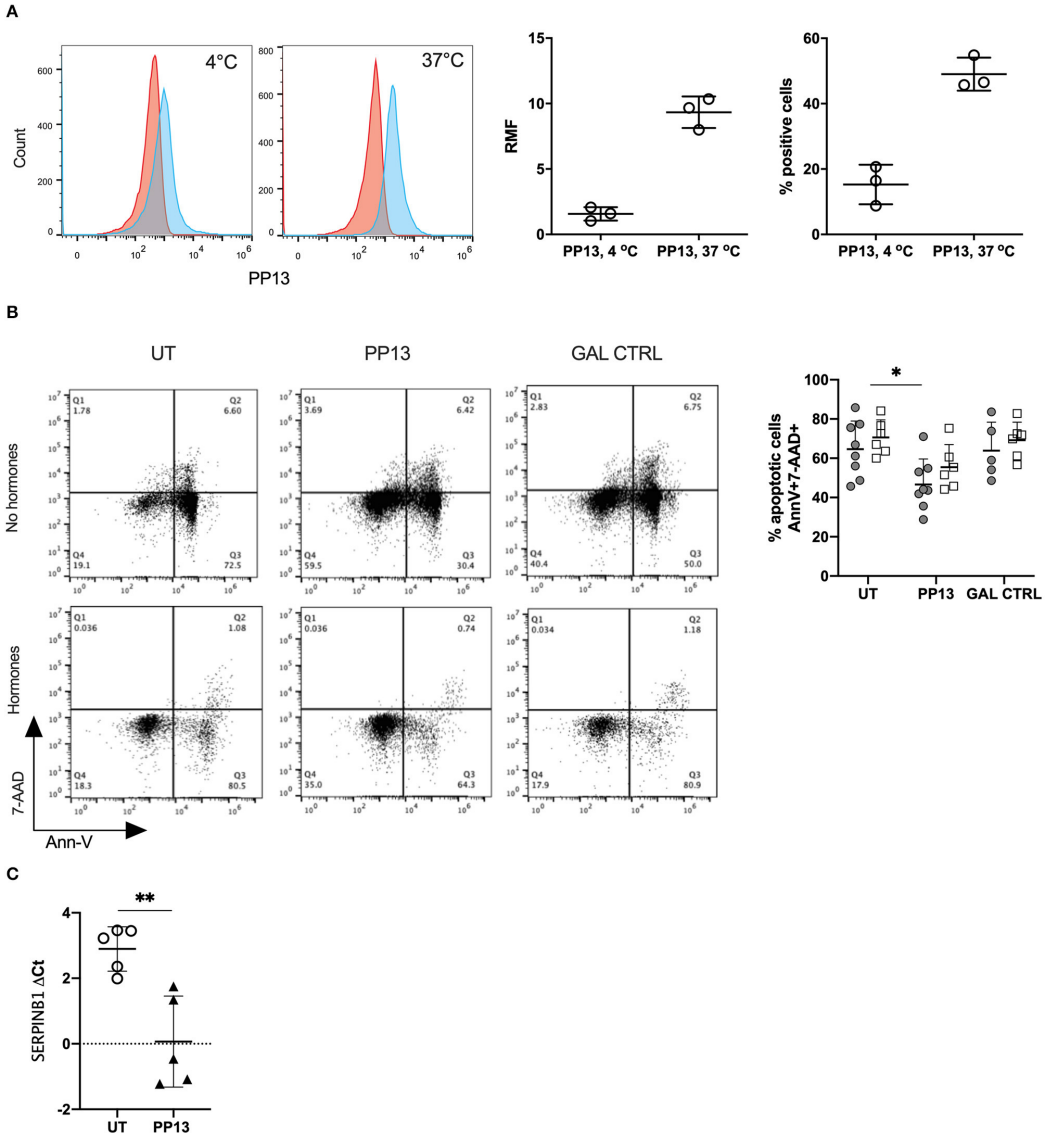
PP13 binds to neutrophils and increases their survival. **(A)** PP13 binding to freshly isolated neutrophils at 4°C and 37°C for 60 min. Flow cytometry plot analysis (red: control, blue: treated with PP13) is represented as the relative mean of fluorescence (RMF) and percentage of positive cells (*n* = 3). **(B)** Apoptosis of neutrophils represented as AnnexinV^+^ and 7-AAD^+/−^ cells after 24 h incubation with PP13 (3 μg/ml) or control Gal (3 μg/ml), in the presence (empty squares, *n* = 6) or absence (full circles, *n* = 8) of pregnancy hormones (progesterone, estradiol, estriol). One way-ANOVA, unpaired samples, ^*^p < 0.05. **(C)** Taqman assay for the expression of *SERPINB1* mRNA in neutrophils after overnight culture with or without PP13 (3 μg/ml) (*n* = 5). Data are presented as ΔCt values (normalization to *GAPDH*). Student's *t*-test, ^**^p < 0.01.

We first investigated if PP13 could bind to the surface of neutrophils since no known receptor for PP13 was described. Indeed, PP13 did bind to the surface of neutrophils represented by relative mean fluorescence (RMF) and the percentage of PP13 positive cells. Both values were higher when neutrophils were incubated at 37°C and not 4°C (RMF: 1.57 ± 0.30 and 9.33 ± 0.70; percentage of positive cells: 49.0 ± 2.9 and 15.3 ± 3.5, respectively) (Figure 1A). We then proceeded to study the ability of PP13 to induce apoptosis in neutrophils since this was recently recognized to be the effect on T cells (9, 40). However, we were surprised to observe that neutrophils in culture with PP13 did survive better and their apoptotic capacity was reduced compared to untreated neutrophils (Figure 1B, gray circles). Since in the decidua neutrophils are also exposed to pregnancy hormones we supplemented culture media with progesterone and estrogen at concentrations mimicking the 3rd trimester. The addition of pregnancy hormones did not modify the response to PP13 for any condition we cultured neutrophils in (Figure 1B, clear squares). Looking for a mechanism contributing to reduced apoptotic capacity, we studied the expression of SerpinB1. SerpinB1, also known as monocyte NE inhibitor is expressed at high levels in the cytoplasm of neutrophils and is one of the most potent inhibitors of NE, cathepsin-G, and proteinase-3, which release from granules during the apoptotic cascade (41). Indeed, PP13 increased the *de novo* RNA transcription of *SERPINB1* in neutrophils (Figure 1C).

### PP13 Does Not Influence the Functionality of Neutrophils

**Figure 2:**
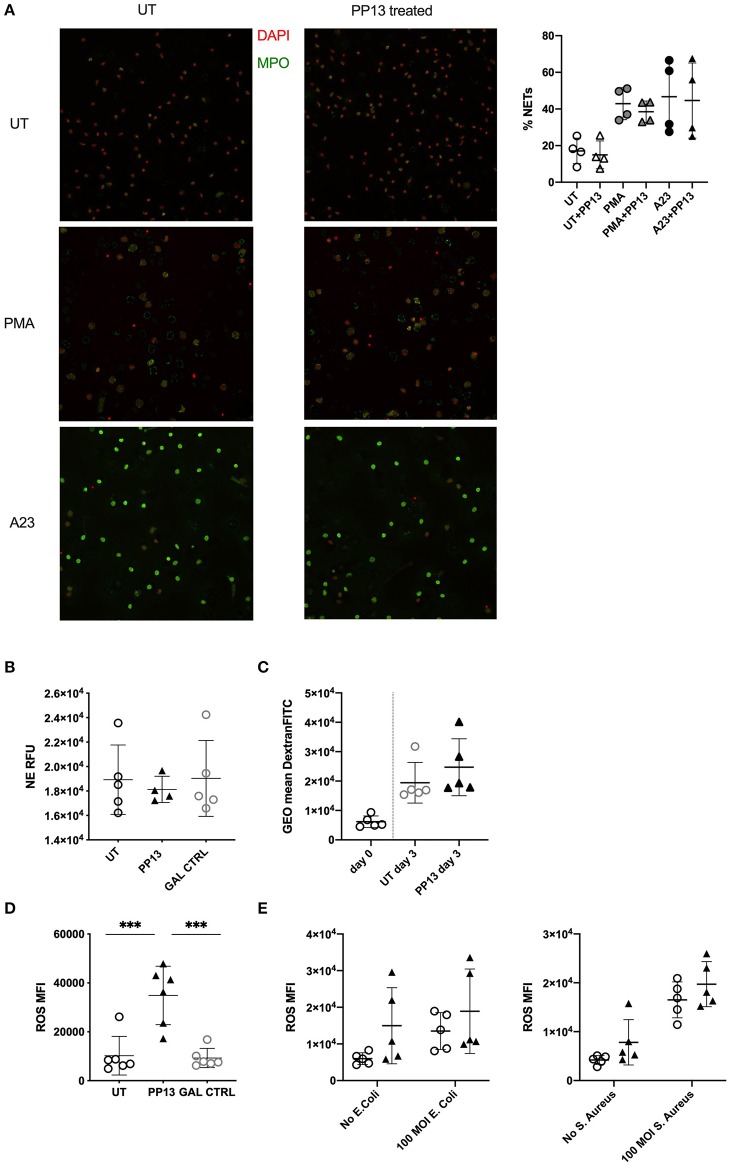
PP13 does not influence the functionality of neutrophils. **(A)** Spontaneous or induced NET formation after 2 h stimulation of neutrophils with or without PP13 (3 μg/ml) and 1 h ± PMA or A23187 (A23) was quantified by immunostaining of neutrophils (*n* = 4). The quantification analysis was performed by NETQUANT. **(B)** NE activity assay performed on supernatants from neutrophils cultured in the presence (*n* = 4) or absence (*n* = 5) of PP13 (3 μg/ml) or control Gal (*n* = 5) for 24 h. **(C)** Phagocytosis of FITC-dextran measured with flow cytometry in neutrophils cultured in the presence or absence of PP13 (3 μg/ml) for 3 days and exposed to FITC-dextran for 60 min (*n* = 5). **(D)** Production of ROS by neutrophils cultured with PP13 (3 μg/ml) or a control Galectin for 24 h (*n* = 6). **(E)** ROS production by neutrophils stimulated with PP13 (3 μg/ml) (triangles) for 30 min with (triangles) or without (circles) heat-killed *E. coli* or *S. aureus* (*n* = 5). All analysis was performed with One-way ANOVA unpaired test, ^***^p < 0.001.

Better surviving neutrophils could constitute a danger for the placental environment if their functionality increases because of protease-mediated tissue damage, or could develop an insufficient barrier if their functionality would be decreased. Therefore, we investigated their ability to spontaneously secrete NETs or upon stimulation with PMA and calcium ionophore (A23187). The presence of PP13 in the culture did not modify the ability of neutrophils to form NETs spontaneously, nor in the presence of stimulants such as PMA or A23187 (Figure 2A). Next, we studied the degranulation ability of neutrophils and used a NE activity assay, where we measured in culture the amount of N-methoxysuccinyl-Ala-Ala-Pro-Val-7-amido-4-methylcoumarin that gets cleaved by NE. The presence of PP13 did not modify the ability to secrete an active form of NE (Figure 2B). Subsequently, we studied the ability to phagocytose using FITC-dextran labeled molecules. Also, after 3 days in culture, PP13 did not modify the ability of neutrophils to phagocytose (Figure 2C). One of the essential functions of neutrophils is to neutralize bacteria via the secretion of reactive oxygen species (ROS). We therefore cultured neutrophils in the presence or absence of PP13 and as control a misfolded galectin. PP13 was able to stimulate an increased release of ROS (Figure 2D). Then, we asked if the presence of bacterial products further stimulated this ability. Neutrophils were hence stimulated with heat-killed *E. coli* (Figure 2E, left panel) or heat-killed *S. aureus* (Figure 2E, right panel). In both cases, we measured that PP13 on its own was able to increase the concentration of ROS in culture and bacterial stimuli did not further increase this capability.

### PP13 Polarizes Neutrophils Toward a Regulatory Phenotype

**Figure 3:**
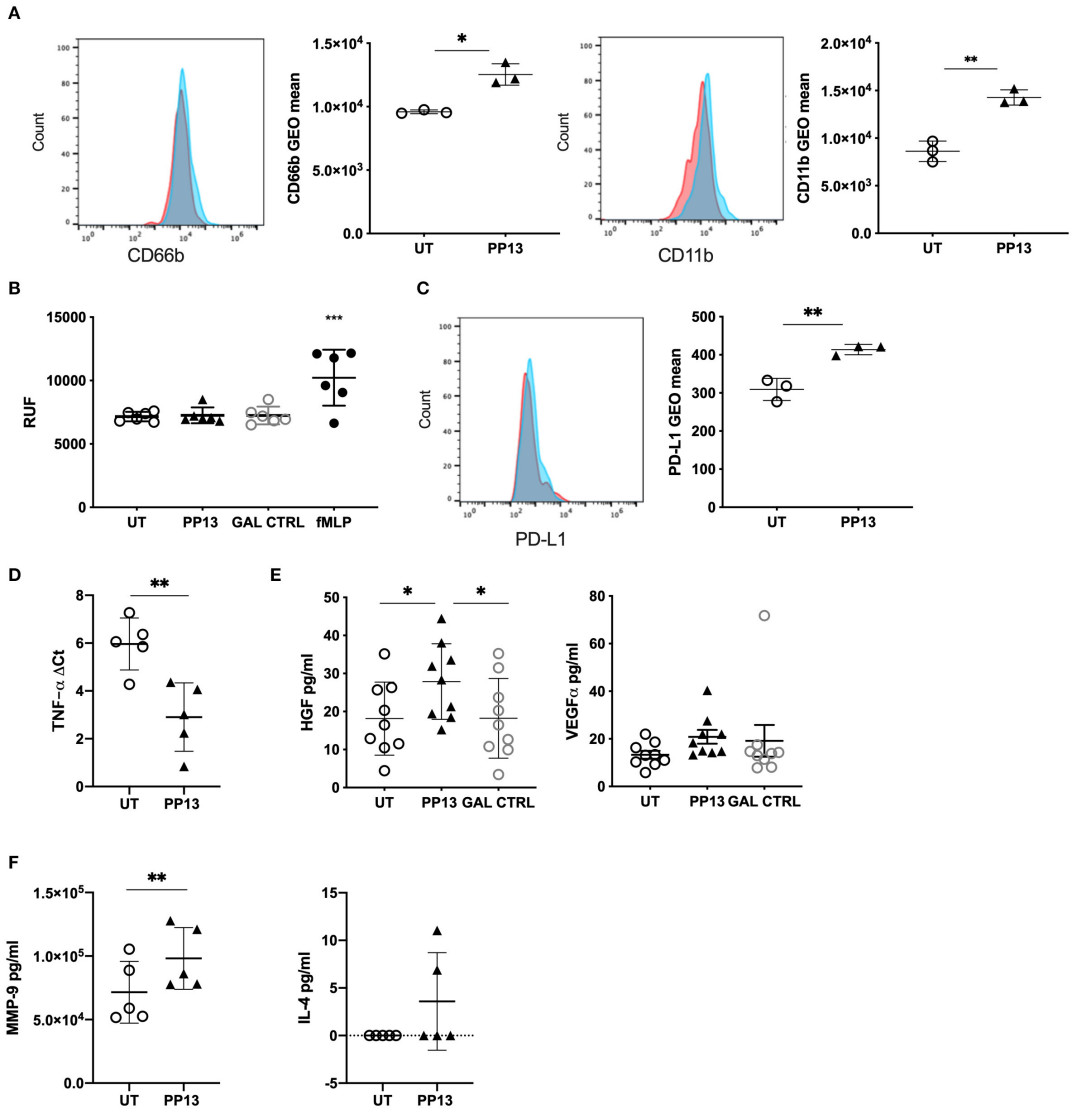
PP13 polarizes neutrophils toward a regulatory phenotype. **(A)** Expression of CD66 or CD11b on the surface of neutrophils co-cultured with BeWo cells and exposed to PP13 (3 μg/ml) (*n* = 3) or left untreated (*n* = 3) for 24 h (red: control, blue: treated with PP13). This is a representative flow cytometry experiment. **(B)** Migration assay of neutrophils in a transwell plate toward PP13, control Gal, or fMLP. **(C)** Expression of PD-L1 on the surface of neutrophils co-cultured with BeWo cells and exposed to PP13 (3 μg/ml) or left untreated for 24 h (*n* = 3) (red: control, blue: treated with PP13). This is a representative flow cytometry experiment. **(D)** *De novo* synthesis of *TNFA* mRNA in neutrophils cultured with or without PP13 for 6 h (*n* = 6). Data are presented as ΔCt values (normalization to *GAPDH*). **(E)** ELISA assay for the secretion of HGF or VEGF-α by neutrophils treated with PP13 (3 μg/ml) or Control Gal for 6 h (*n* = 9). **(F)** ELISA assay for the secretion of MMP-9 or IL-4 by neutrophils treated with PP13 (3 μg/ml) for 6 h (*n* = 5) (^**^p < 0.01). All analysis was performed with Student's *t*-test (number of groups are 2) or One-way ANOVA unpaired test (number of groups are more than 2), ^*^p < 0.05, ^**^p < 0.01, ^***^p < 0.001.

Since PP13 did not modify neutrophil functionality, we wondered whether their phenotype could result in polarization i.e., like in cancer tissue. The placenta, and more specifically, the decidua, is a very particular environment that requires to keep tolerance toward the fetus while maintaining the functionality of the adaptive and the innate immune system. Thus, we studied the surface expression of adhesion molecule CD66b (Figure 3A, left panel) and integrin CD11b (Figure 3B, right panel) on neutrophils co-cultured with BeWo cells, a very simplified method to mimick the trophoblast-maternal immune cell contacts in the decidua. In both cases, the presence of PP13 increased the expression of both molecules. Similarly, PP13 stimulation of neutrophils in absence of BeWo cells increased the expression of both molecules (Supplemental Figures 1A,B). We investigated if the increased presence of CD66b and CD11b did increase the ability of neutrophils to migrate. In a transwell assay we could observe that PP13 did not affect the ability of neutrophils to migrate (Figure 3B).

The molecule that the mostly characterizes regulatory molecules in cancer is PD-L1. We, therefore studied its expression on neutrophils exposed to PP13 and co-cultured on BeWo. PD-L1 was upregulated on neutrophils (Figure 3C) when exposed to PP13 suggesting the possibility of a regulatory phenotype. Similarly, PP13 stimulation of neutrophils in absence of BeWo cells increased the surface expression of PD-L1 (Supplemental Figure 1C). Since TNF-α is a potent multifunctional cytokine in autocrine and paracrine processes and is central to reproduction and placental differentiation (42), we studied the ability to produce *de novo* TNF-α in neutrophils exposed to PP13. Indeed, we could measure an increase in RNA transcription for TNF-α (Figure 3D). We investigated if neutrophils were also able to produce other growth factors that are able to influence the growth of the placenta including HGF (27, 28) and VEGF-α. Indeed, PP13 increased the secretion of HGF (Figure 3E, right panel) from neutrophils. Neutrophils already produced VEGF-α and the exposure to PP13 did not modify its expression (Figure 3E, right panel). Other molecules described to be expressed in neutrophils in the cancer niche are MMP-9, IL-4, and IL-10 (although very controversial). In our case, we could observe that PP13 increased the release of MMP-9 (Figure 3F, left panel) but not of IL-4 (Figure 3F, right panel), which was found to be in the lower detection range of the assay. IL-10 release was not detected either in PP13-treated neutrophils or in the untreated population (data not shown).

## Discussion

Herein, we describe the phenotype and the functionality of neutrophils exposed to recombinant human PP13, a placenta-specific protein *in vivo* produced and secreted by the syncytiotrophoblast. In a concentration similar to the one measured in placenta, PP13 shifted the neutrophils phenotype toward an immunoregulatory phenotype similar to the one observed in cancer. Here neutrophils express molecules to sustain trophoblast growth and angiogenesis, to control T cell activity while maintaining their capacity to respond to bacteria, to degranulate, to produce NET, and to phagocytose. We observed that neutrophils cultured with PP13, in the presence or absence of pregnancy hormones do not undergo apoptosis as previously described for T cells (9). We questioned if neutrophils switch their ability to undergo apoptosis spontaneously. We observed that the *de novo* expression of the *SERPINB1* an essential regulator of proteases cathepsin G and proteinase-3 that mediate granule release and death pathways in neutrophils was increased (41).

Since neutrophil functionality in the placenta is of primary importance to control infections and previously it was described that trophoblast cells when in direct contact with neutrophils are able to modify the calcium response of neutrophils and their activation profile via pyruvate (43). Hence, we studied the most common functions of neutrophils. We could observe that PP13 did not interfere with spontaneous NET release, degranulation, phagocytosis and bacterial-ROS-response. However, PP13 was very effective in inducing the production of ROS. This ability was described to have different functions. One of these is controlling T cell proliferation upon activation and was described in several immunological settings [as reviewed in (44)]. Thus, we speculated that PP13 could polarize neutrophils toward a “placental-growth-permissive” phenotype, which should resemble the one described in the cancer setting. Therefore, we studied the phenotype and growth factor expression upon stimulation with PP13. Indeed, we could confirm the expression of CD66b, CD11b, and PD-L1, HGF and VEGF, MMP-9 and TNF-α. We observed the expression of PD-L1 on neutrophils; a ligand already described to interfere with T cell activity (21). TNF-α was already recognized early on as being a potent multifunctional cytokine in an autocrine and paracrine process central to reproduction and essential for trophoblast differentiation (42).

Additionally, TNF-α, although not alone, was capable of upregulating MET (receptor for HGF) expression and promoting the antitumor activity of neutrophils in a variety of cancer types (45). Neutrophils were already shown to produce HGF while infiltrating in bronchioloalveolar adenocarcinoma (46) and we measured an increased secretion of HGF if neutrophils were exposed to PP13. Since its receptor mediates the biological effects of HGF (c-Met) a transmembrane protein encoded by the *MET* proto-oncogene (47) and the receptor for HGF is localized in placental cytotrophoblasts and the syncytiotrophoblast (28), we proposed that the expression of this growth factor is important for the growth regulation of the trophoblast. Indeed, the knockout of HGF in mice is embryonically lethal due to impaired organogenesis of the placenta and liver (46) with markedly reduced number of labyrinthine trophoblasts in the placenta. It is noteworthy that HGF-Met participates in a long-distance migration of cells in development, which indicates a particular role for HGF in cell movement (48). Interestingly, the role of MMP-9 in the placenta is recognized to loosen the tight junctions and regulate the invasion of endothelial cells to promote spiral artery remodeling (49). At the same time, MMPs are also capable of remodeling the extracellular matrix to promote angiogenesis (25).

We recognize the following limitations of the study: (1) we reduced the complexity of a placental system to a 1 or 2 component system plus treatment. This oversimplification of the placental-system is useful to understand the direct effect of PP13 on neutrophils but does not take into account the role of other immune players or mesenchymal/endothelial cells in further shaping the phenotype of neutrophils. Therefore, we aim to study this matter in the placenta. However, it is extremely challenging to obtain placental tissue from early healthy pregnancies; (2) human neutrophils from non-pregnant donors were used to study the effect of PP13 since blood circulating neutrophils isolated from pregnant women are already exposed to PP13 and different hormone concentrations and could already be modified in their biological response. Thus, we decided to use non-exposed neutrophils; (3) pregnancy hormones were taken into consideration only for the apoptosis experiment. Since PP13 did not influence the functionality of neutrophils, we did not pursue the effect of this variable; (4) another critical point to discuss is the concentration of PP13 used for the study; this matched the calculation of the concentration measured in human placenta at the end of the second through to the third trimester (50). Unfortunately, no data are available on placental PP13 concentrations at early stages of gestation; (5) The BeWo cell line is derived from first trimester choriocarcinoma cells instead of primary trophoblast for the co-culture experiments, since primary trophoblast cells are challenging to obtain. We are aware that this does not completely represent trophoblast cells.

Taken together, in this study, we describe the polarizing effect of a placenta- specific galectin on neutrophils. Since placenta and tumors share common features, i.e., invasiveness, high degrees of cell turnover, requirement for angiogenesis, immune regulation to suppress the adaptive immune response to allo-/tumor-antigens, we propose that PP13 could shift neutrophils toward a placental-growth-permissive phenotype, recalling the one observed in cancer while maintaining all their primary functions and abilities to respond to bacterial invasion.

## Data Availability Statement

The datasets generated for this study are available on request to the corresponding author.

## Ethics Statement

The studies involving human participants were reviewed and approved by Ethikkommision Nordwest- und Zentralschweiz. The patients/participants provided their written informed consent to participate in this study.

## Author Contributions

LV, SV, GS, AB, and ET performed experiments. NGT provided materials, discussed data, and contributed to the writing of the manuscript. LV, SH, and SR conceived the experiments. LV and SR wrote the manuscript. LV prepared the figures. All authors discussed and commented the data and the manuscript.

### Conflict of Interest

The authors declare that the research was conducted in the absence of any commercial or financial relationships that could be construed as a potential conflict of interest.

## References

[1] Pollheimer J Vondra S Baltayeva J Beristain AG Knöfler M. Regulation of placental extravillous trophoblasts by the maternal uterine environment. Front Immunol. (2018) 9:297–18. 10.3389/fimmu.2018.02597 30483261 PMC6243063

[2] Than NG Sumegi B Than GN Berente Z Bohn H. Isolation and sequence analysis of a cDNA encoding human placental tissue protein 13 (PP13), a new lysophospholipase, homologue of human eosinophil Charcot-Leyden Crystal protein. Placenta. (1999) 20:703–10. 10.1053/plac.1999.0436 10527825

[3] Than NG Romero R Xu Y Erez O Xu Z Bhatti G. Evolutionary origins of the placental expression of chromosome 19 cluster galectins and their complex dysregulation in preeclampsia. Placenta. (2014) 35:855–65. 10.1016/j.placenta.2014.07.015 25266889 PMC4203431

[4] Burger O Pick E Zwickel J Klayman M Meiri H Slotky R. Placental protein 13 (PP-13): effects on cultured trophoblasts, and its detection in human body fluids in normal and pathological pregnancies. Placenta. (2004) 25:608–22. 10.1016/j.placenta.2003.12.009 15193867

[5] Huppertz B Sammar M Chefetz I Neumaier-Wagner P Bartz C Meiri H. Longitudinal determination of serum placental protein 13 during development of preeclampsia. Fetal Diagn Ther. (2008) 24:230–6. 10.1159/000151344 18753763

[6] Than NG Romero R Tarca AL Kekesi KA Xu Y Xu Z. Integrated systems biology approach identifies novel maternal and placental pathways of preeclampsia. Front Immunol. (2018) 9:159. 10.3389/fimmu.2018.01661 30135684 PMC6092567

[7] Than NG Romero R Goodman M Weckle A Xing J Dong Z. A primate subfamily of galectins expressed at the maternal-fetal interface that promote immune cell death. Proc Natl Acad Sci USA. (2009) 106:9731–6. 10.1073/pnas.0903568106 19497882 PMC2689813

[8] Muro P Capobianco G Lepedda AJ Nieddu G Formato M Tram NHQ. Plasma PP13 and urinary GAGs/PGs as early markers of pre-eclampsia. Arch Gynecol Obstetr. (2016) 294:959–65. 10.1007/s00404-016-4111-0 27161490

[9] Balogh A Toth E Romero R Parej K Csala D Szenasi NL. Placental galectins are key players in regulating the maternal adaptive immune response. Front Immunol. (2019) 10:1240. 10.3389/fimmu.2019.01240 31275299 PMC6593412

[10] Gupta AK Hasler P Holzgreve W Gebhardt S Hahn S. Induction of neutrophil extracellular DNA lattices by placental microparticles and IL-8 and their presence in preeclampsia. Hum Immunol. (2005) 66:1146–54. 10.1016/j.humimm.2005.11.003 16571415

[11] Stoikou M Grimolizzi F Giaglis S Schäfer G van Breda SV Hoesli IM. Gestational diabetes mellitus is associated with altered neutrophil activity. Front Immunol. (2017) 8:1749. 10.3389/fimmu.2017.00702 28659928 PMC5469883

[12] Gomez-Lopez N Romero R Leng Y Garcia-Flores V Xu Y Miller D. Neutrophil extracellular traps in acute chorioamnionitis: a mechanism of host defense. Am J Reprod Immunol. (2017) 77:1–10. 10.1111/aji.12617 28045214 PMC5370569

[13] Gomez-Lopez N Romero R Garcia-Flores V Xu Y Leng Y Alhousseini A. Amniotic fluid neutrophils can phagocytize bacteria: a mechanism for microbial killing in the amniotic cavity. Am J Reprod Immunol. (2017) 78:e12723. 10.1111/aji.12723 28703488 PMC5623137

[14] Mor G Aldo P Alvero AB The unique immunological and microbial aspects of pregnancy. Nat Rev Immunol. (2017) 7:320–482. 10.1038/nri.2017.64 28627518

[15] Amsalem H Kwan M Hazan A Zhang J Jones RL Whittle W. Identification of a novel neutrophil population: proangiogenic granulocytes in second-trimester human decidua. J Immunol. (2014) 193:3070–9. 10.4049/jimmunol.1303117 25135830

[16] Croxatto D Micheletti A Montaldo E Orecchia P Loiacono F Canegallo F. Group 3 innate lymphoid cells regulate neutrophil migration and function in human decidua. Mucosal Immunol. (2016) 9:1372–83. 10.1038/mi.2016.10 26906405

[17] Vacca P Vitale C Munari E Cassatella MA Mingari MC Moretta L. Human innate lymphoid cells: their functional and cellular interactions in decidua. Front Immunol. (2018) 9:1897. 10.3389/fimmu.2018.01897 30154799 PMC6102343

[18] Kliman HJ Sammar M Grimpel YI Lynch SK Milano KM Pick E. Placental protein 13 and decidual zones of necrosis: an immunologic diversion that may be linked to preeclampsia. Reprod Sci. (2012) 19:16–30. 10.1177/1933719111424445 21989657

[19] Giese MA Hind LE Huttenlocher A. Neutrophil plasticity in the tumor microenvironment. Blood. (2019) 133:2159–67. 10.1182/blood-2018-11-844548 30898857 PMC6524564

[20] Wu L Saxena S Awaji M Singh RK. Tumor-associated neutrophils in cancer: going pro. Cancers. (2019) 11:564. 10.3390/cancers11040564 31010242 PMC6520693

[21] Cheng Y Li H Deng Y Tai Y Zeng K Zhang Y. Cancer-associated fibroblasts induce PDL1+ neutrophils through the IL6-STAT3 pathway that foster immune suppression in hepatocellular carcinoma. Cell Death Dis. (2018) 9:422. 10.1038/s41419-018-0458-4 29556041 PMC5859264

[22] Kasten KR Muenzer JT Caldwell CC. Neutrophils are significant producers of IL-10 during sepsis. Biochem Biophys Res Commun. (2010) 393:28–31. 10.1016/j.bbrc.2010.01.066 20097159 PMC2830356

[23] Brandt E Woerly G Ben Younes A Loiseau S Capron M. IL-4 production by human polymorphonuclear neutrophils. J Leukoc Biol. (2000) 68:125–130. 10.1189/jlb.68.1.125 10914499

[24] Siwetz M Blaschitz A El-Heliebi A Hiden U Desoye G Huppertz B. TNF-α alters the inflammatory secretion profile of human first trimester placenta. Lab Invest. (2016) 96:428–38. 10.1038/labinvest.2015.159 26752743

[25] Ardi VC Kupriyanova TA Deryugina EI Quigley JP. Human neutrophils uniquely release TIMP-free MMP-9 to provide a potent catalytic stimulator of angiogenesis. Proc Natl Acad Sci USA. (2007) 104:20262–7. 10.1073/pnas.0706438104 18077379 PMC2154419

[26] McCourt M Wang JH Sookhai S Redmond HP. Proinflammatory mediators stimulate neutrophil-directed angiogenesis. Arch Surg. (1999) 134:1325–31. 10.1001/archsurg.134.12.1325 10593330

[27] Kauma S Hayes N Weatherford S. The differential expression of hepatocyte growth factor and met in human placenta. J Clin Endocrinol Metab. (1997) 82:949–54. 10.1210/jc.82.3.949 9062512

[28] Somerset DA Li X-F Afford S Strain AJ Ahmed A Sangha RK. Ontogeny of hepatocyte growth factor (HGF) and its receptor (c-met) in human placenta. Am J Pathol. (2010) 153:1139–47. 10.1016/S0002-9440(10)65658-1 9777945 PMC1853066

[29] Ushio-Fukai M Nakamura Y. Reactive oxygen species and angiogenesis: NADPH oxidase as target for cancer therapy. Cancer Lett. (2008) 266:37–52. 10.1016/j.canlet.2008.02.044 18406051 PMC2673114

[30] Cemerski S Cantagrel A van Meerwijk JPM Romagnoli P. Reactive oxygen species differentially affect T cell receptor-signaling pathways. J Biol Chem. (2002) 277:19585–93. 10.1074/jbc.M111451200 11916964

[31] Kapust RB Waugh DS. Controlled intracellular processing of fusion proteins by TEV protease. Protein Expr Purif. (2000) 19:312–8. 10.1006/prep.2000.1251 10873547

[32] Nekrasova IV Shirshev SV. Female sex steroid hormones in regulation of neutrophil enzymatic activity. Dokl Biochem Biophys. (2013) 453:312–5. 10.1134/S1607672913060100 24385104

[33] Fuhler GM Blom NR Coffer PJ Drayer AL Vellenga E. The reduced GM-CSF priming of ROS production in granulocytes from patients with myelodysplasia is associated with an impaired lipid raft formation. J Leukoc Biol. (2007) 81:449–57. 10.1189/jlb.0506311 17079651

[34] Deng X Ueda H Su SB Gong W Dunlop NM Gao JL. A synthetic peptide derived from human immunodeficiency virus type 1 gp120 downregulates the expression and function of chemokine receptors CCR5 and CXCR4 in monocytes by activating the 7-transmembrane G-protein-coupled receptor FPRL1/LXA4R. Blood. (1999) 94:1165–73. 10.1182/blood.V94.4.1165 10438703

[35] Auvynet C Moreno S Melchy E Coronado-Martínez I Montiel JL Aguilar-Delfin I. Galectin-1 promotes human neutrophil migration. Glycobiology. (2013) 23:32–42. 10.1093/glycob/cws128 22942212

[36] Rochael NC Guimarães-Costa AB Nascimento MTC DeSouza-Vieira TS Oliveira MP Souza LFGE. Classical ROS-dependent and early/rapid ROS-independent release of neutrophil extracellular traps triggered by leishmania parasites. Nat Publish Group. (2015) 5:1–11. 10.1038/srep18302 26673780 PMC4682131

[37] Sur Chowdhury C Giaglis S Walker UA Buser A Hahn S Hasler P. Enhanced neutrophil extracellular trap generation in rheumatoid arthritis: analysis of underlying signal transduction pathways and potential diagnostic utility. Arthritis Res Ther. (2014) 16:R122. 10.1186/ar4579 24928093 PMC4229860

[38] Giaglis S Stoikou M Chowdhury CS Schaefer G Grimolizzi F Rossi SW. Multimodal regulation of NET formation in pregnancy: progesterone antagonizes the Pro-NETotic effect of estrogen and G-CSF. Front Immunol. (2016) 7:565. 10.3389/fimmu.2016.00565 27994595 PMC5136684

[39] Mohanty T Sørensen OE Nordenfelt P. NETQUANT: automated quantification of neutrophil extracellular traps. Front Immunol. (2018) 8:3503. 10.3389/fimmu.2017.01999 29379509 PMC5775513

[40] Than NG Balogh A Romero R Karpati E Erez O Szilagyi A. Placental protein 13 (PP13) - a placental immunoregulatory galectin protecting pregnancy. Front Immunol. (2014) 5:348. 10.3389/fimmu.2014.00348 25191322 PMC4138504

[41] Baumann M Pham CTN Benarafa C. SerpinB1 is critical for neutrophil survival through cell-autonomous inhibition of cathepsin G. Blood. (2013) 121:3900–7, S1–6. 10.1182/blood-2012-09-455022 23532733 PMC3650706

[42] Terranova PF Hunter VJ Roby KF Hunt JS. Tumor necrosis factor-alpha in the female reproductive tract. Proc Soc Exp Biol Med. (1995) 209:325–42. 10.3181/00379727-209-43905B 7638240

[43] Petty HR Kindzelskii AL Espinoza J Romero R. Trophoblast contact deactivates human neutrophils. J Immunol. (2006) 176:3205–14. 10.4049/jimmunol.176.5.3205 16493081

[44] Leliefeld PHC Koenderman L Pillay J. How neutrophils shape adaptive immune responses. Front Immunol. (2015) 6:471. 10.3389/fimmu.2015.00471 26441976 PMC4568410

[45] Finisguerra V Di Conza G Di Matteo M Serneels J Costa S Thompson AAR. MET is required for the recruitment of anti-tumoural neutrophils. Nature. (2015) 522:349–53. 10.1038/nature14407 25985180 PMC4594765

[46] Wislez M Rabbe N Marchal J Milleron B Crestani B Mayaud C. Hepatocyte growth factor production by neutrophils infiltrating bronchioloalveolar subtype pulmonary adenocarcinoma: role in tumor progression and death. Cancer Res. (2003) 63:1405–12. 12649206

[47] Bottaro DP Rubin JS Faletto DL Chan AM Kmiecik TE Vande Woude GF. Identification of the hepatocyte growth factor receptor as the c-met proto-oncogene product. Science. (1991) 251:802–4. 10.1126/science.1846706 1846706

[48] Ueno M Lee LK Chhabra A Kim YJ Sasidharan R Van Handel B. c-Met-dependent multipotent labyrinth trophoblast progenitors establish placental exchange interface. Dev Cell. (2013) 27:373–86. 10.1016/j.devcel.2013.10.019 24286824 PMC3950757

[49] Espino Y Sosa S Flores-Pliego A Espejel-Nuñez A Medina-Bastidas D Vadillo-Ortega F Zaga-Clavellina V New insights into the role of matrix metalloproteinases in preeclampsia. IJMS. (2017) 18:1448–10. 10.3390/ijms18071448 PMC5535939 28726716

[50] Than NG Abdul Rahman O Magenheim R Nagy B Fule T Hargitai B. Placental protein 13 (galectin-13) has decreased placental expression but increased shedding and maternal serum concentrations in patients presenting with preterm pre-eclampsia and HELLP syndrome. Virchows Arch. (2008) 453:387–400. 10.1007/s00428-008-0658-x 18791734 PMC2775473

