# Supplementary figures and images for: Placental Protein 13 (Galectin-13) Polarizes Neutrophils Toward an Immune Regulatory Phenotype
Source: Front Immunol. 2020 Feb 12;11:145. doi: 10.3389/fimmu.2020.00145 (PMC7028707; doi:10.3389/fimmu.2020.00145)

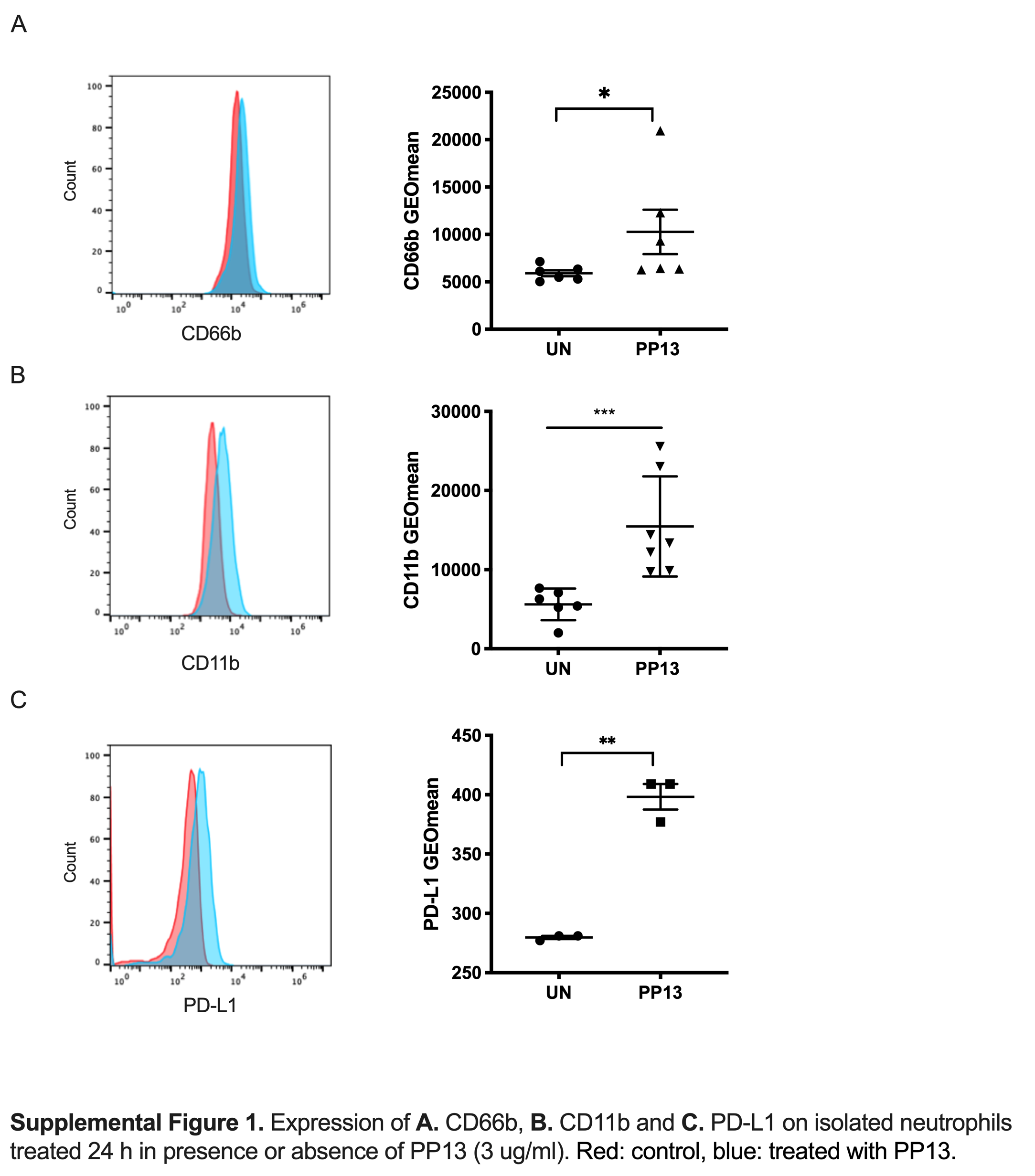

Supplement: Supplementary file 1 [file Image_1.TIFF]
